# Inhibition of Kv1.3 Channels in Human Jurkat T Cells by Xanthohumol and Isoxanthohumol

**DOI:** 10.1007/s00232-015-9782-0

**Published:** 2015-02-17

**Authors:** Justyna Gąsiorowska, Andrzej Teisseyre, Anna Uryga, Krystyna Michalak

**Affiliations:** Department of Biophysics, Wroclaw Medical University, ul. Chałubińskiego 10, 50-368 Wrocław, Poland

**Keywords:** Prenylated flavonoids, Prenylated chalcones, Kv1.3 channel, Jurkat T cells, Cancer cell proliferation, Cancer cell apoptosis

## Abstract

Using whole-cell patch-clamp technique, we investigated influence of selected compounds from groups of prenylated chalcones and flavonoids: xanthohumol and isoxanthohumol on the activity of Kv1.3 channels in human leukemic Jurkat T cells. Obtained results provide evidence that both examined compounds were inhibitors of Kv1.3 channels in these cells. The inhibitory effects occurred in a concentration-dependent manner. The estimated value of the half-blocking concentration (EC_50_) was about 3 μM for xanthohumol and about 7.8 μM for isoxanthohumol. The inhibition of Kv1.3 channels by examined compounds was not complete. Upon an application of the compounds at the maximal concentrations equal to 30 μM, the activity of Kv1.3 channels was inhibited to about 0.13 of the control value. The inhibitory effect was reversible. The application of xanthohumol and isoxanthohumol did not change the currents’ activation and inactivation rate. These results may confirm our earlier hypothesis that the presence of a prenyl group in a molecule is a factor that facilitates the inhibition of Kv1.3 channels by compounds from the groups of flavonoids and chalcones. The inhibition of Kv1.3 channels might be involved in antiproliferative and proapoptotic effects of the compounds observed in cancer cell lines expressing these channels.

## Introduction

Voltage-gated potassium channels of the Kv1.3 type are widely expressed in many types of cells, both normal and cancer (Felipe et al. 2006; Gutman et al. 2005). Activity of Kv1.3 channels plays an important role e.g. in setting the resting membrane potential, cell proliferation, apoptosis and volume regulation (Cahalan and Chandy 2009; Gulbins et al. 2010). Studies performed on Kv1.3 channels expressed in human T lymphocytes showed that blockage of these channels inhibited the cell proliferation in the G_1_ phase (Cahalan and Chandy 2009). Specific blockers of Kv1.3 channels may be applied in a selective immunosuppression (Cahalan and Chandy 2009).

Several studies demonstrated altered expression of Kv1.3 channels in some cancer diseases such as breast, colon, pancreas, smooth muscle, skeletal muscle, lung, kidney and prostate cancer (Bielanska et al. 2009; Comes et al. 2013). Inhibitors of Kv1.3 channels may potentially find a clinical application in therapy of some cancer disorders characterized by an over-expression of Kv1.3 channels, such as, for example, breast and lung cancer, melanoma or chronic lymphocytic leukaemia (Jang et al. 2009, 2011; Leanza et al. 2012, 2013). For example, it was shown that inhibition of Kv1.3 channels by both specific (MgTX) and non-specific (TEA) inhibitors also inhibited proliferation of breast cancer (Jang et al. 2009) and lung cancer cells in vitro and in vivo (Jang et al. 2011). Among many inhibitors of Kv1.3 channels, the most promising candidates for a potential clinical application may be some small molecule organic compounds, since they combine a high efficiency and specificity of cancer cell elimination with a good bioavailability and a low cytotoxity (Leanza et al. 2012, 2013). One of these compounds, clofazimine, is widely applied in medicine (in a treatment of leprosy, for example) since 1960s’ (Leanza et al. 2012, 2013).

Studies performed during last years in our laboratory showed that both some natural plant-derived compounds that exert antiproliferative and proapoptotic effects on cancer cells, such as an isoflavone genistein, substituted stilbene, resveratrol, and some synthetic methoxy derivatives of a substituted stilbene, piceatannol and of a flavonoid, naringenin, were inhibitors of Kv1.3 channels in normal human T lymphocytes (TL) (Teisseyre and Michalak 2005, 2006; Teisseyre et al. 2009). Importantly, the antiproliferative effect on cancer cells and inhibition of Kv1.3 channels occurred at comparable concentrations of the compounds tested (Teisseyre and Michalak 2005, 2006; Teisseyre et al. 2009). It was concluded that the antiproliferative effect of these compounds could be, at least partially, related to the inhibition of Kv1.3 channels (Teisseyre and Michalak 2005, 2006; Teisseyre et al. 2009). Our previous studies also showed that a plant-derived prenylated flavonoid 8-prenylnaringenin—in contrast to its precursor—naringenin was an effective inhibitor of Kv1.3 channels both in normal human TL and in a human cancer T cell line—Jurkat (Gąsiorowska et al. 2012). The estimated value of a half-blocking concentration (EC_50_) was about 2.5 μM. A complete inhibition occurred at the concentrations higher than 10 μM. It was shown that 8-prenylnaringenin was the most potent inhibitor of Kv1.3 channels from all plant-derived polycyclic compounds tested to date in our laboratory (Gąsiorowska et al. 2012). The potency of the inhibition could be a consequence of the presence of prenyl group in the molecule of this flavonoid. If this hypothesis was correct it could be expected that other compounds from the groups of prenylated flavonoids and chalcones would also be potent inhibitors of Kv1.3 channels.

**Fig. 1:**
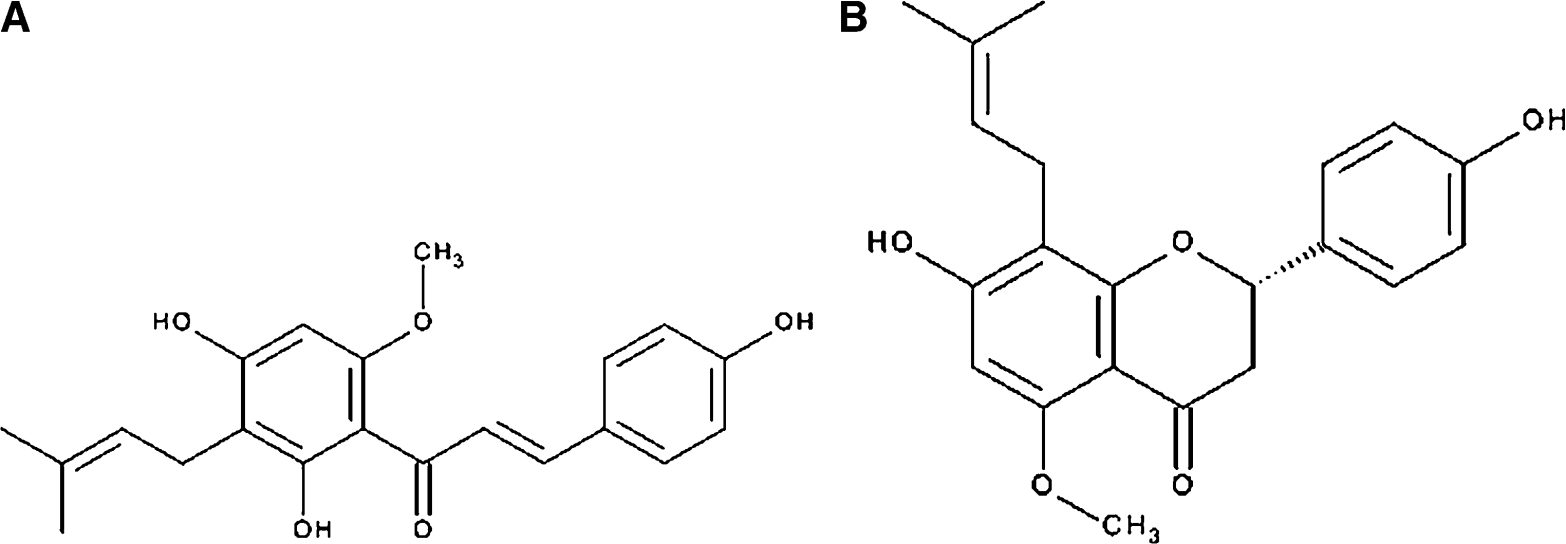
Chemical structures of: xanthohumol (**a**) and isoxanthohumol (**b**)

In this study, we tested other prenylated compounds from both groups: a chalcone: xanthohumol and a flavonoid: isoxanthohumol (Fig. 1 near here).

Xanthohumol (X) is a structurally simple prenylated chalcone that occurs in the hop plant *Humulus lupulus* (0.1–1 % on dry weight) and it is used to add bitterness and flavour to beer, which is the main dietary source of xanthohumol (Stevens and Page 2004). Isoxanthohumol, structurally related to 8-prenylnaringenin, is a product of an isomerization of xanthohumol during the brewing process and it is the main prenylflavonoid in beer (Stevens et al. 1999). Both compounds have focused much attention in recent years as cancer chemopreventive agents. Obtained results provide evidence that both xanthohumol and isoxanthohumol, applied at micromolar concentrations, exert antiproliferative and cytotoxic effects on human breast cancer cell line MCF-7, colon cancer cell line HT-29 and ovarian cancer cell line A-2780 (Miranda et al. 1999). It was also shown that an incubation of xanthohumol and isoxanthohumol applied at micromolar concentrations with prostate cancer cell lines PC-3 and DU145 induced a caspase-independent form of cell death (Delmulle et al. 2008). Moreover, both xanthohumol and isoxanthohumol exert antiangiogenic and antiinflammatory effects on human umbilical vein endothelial cells (HUVEC) and human aortic smooth muscle cells (HASMC) (Negrao et al. 2010).

Since Kv1.3 channels are widely expressed in human leukemic T cell line Jurkat (Attali et al. 1992; Valencia-Cruz et al. 2009), these cells were used in our study as a model system. Obtained results provide evidence that both selected compounds effectively inhibited Kv1.3 channels.

## Materials and Methods

### Cell Culture and Solutions

The human leukemic T cell line, Jurkat (clone E6-1), was purchased from American Type Culture Collection (Manassas, VA). The Jurkat cells were grown in RPMI 1640 medium (Sigma-Aldrich, St. Louis, MO) containing 10 % heat-inactivated FBS, 10 mM HEPES and 2 mM glutamate. Cells were grown on culture plates at 37 °C in a 5 % CO_2_-humidified incubator. During the experiments, cells were placed in the external solution containing, in mM: 150 NaCl, 4.5 KCl, 1 CaCl_2_, 1 MgCl_2_, 10 HEPES and pH 7.35 adjusted with NaOH, 300 mOsm. The pipette solution contained in mM: 150 KCl, 1 CaCl_2_, 2 MgCl_2_, 10 HEPES 10 EGTA, and pH 7.2 was adjusted with KOH, 280 mOsm. The concentration of free calcium ions in the internal solution was below 100 nM, assuming the dissociation constant for EGTA at pH 7.2 of 10^−7^ M (Grissmer et al. 1993). Such a low-calcium concentration was applied to prevent the activation of calcium-activated K^+^ channels K_Ca_2.2 abundantly expressed in Jurkat T cells (Grissmer et al. 1992). The chemicals were purchased from the Polish Chemical Company (POCH, Gliwice, Poland), except of HEPES and EGTA that were purchased from SIGMA. The examined compounds were purchased from Alexis Biochemicals (Lausen, Switzerland).

### Patch-Clamp Recordings

Dishes with cells were placed under an inverted Olympus IMT-2 microscope. Solutions containing tested compounds were applied using a perfusion system developed in our laboratory. Pipettes were pulled from a borosilicate glass (Hilgenberg, Germany) and fire polished before the experiment. The pipette resistance was in the range of 3–5 MΩ.

Whole-cell potassium currents in TL were recorded applying the patch-clamp technique (Hamill et al. 1981). The currents were recorded using an Axopatch 200B Amplifier (Molecular Devices Corp., USA), low-pass filtered at 3 kHz, digitised using Digidata 1440 (Molecular Devices Corp., USA) analogue-to-digital converter with the sampling rate of 10 kHz. The influence of selected compounds on the activity of the channels was studied by applying the voltage ramp protocol. Voltage ramps depolarising cell membranes from −100 mV up to +40 mV were applied every 20 s; the ramp duration was 340 ms and holding potential −90 mV. Upon application of the voltage ramp protocol, potassium currents in Jurkat T cells were stably recorded for at least 20 min after “break-in” to the whole-cell configuration. During the off-line analysis, the value of Kv1.3 current at the end of a voltage ramp (+40 mV) was calculated. For this purpose, the leak current estimated at +40 mV was subtracted from the total ramp current recorded at this voltage. The calculated voltage-clamp error, due to an uncompensated access resistance, was 4.0 ± 0.5 mV (*n* = 30).

All experiments were carried out at room temperature (22–24 °C).

The data are presented as mean ± standard error.

### Data Analysis

The inhibition of the Kv1.3 channels is presented in terms of a relative current recorded upon application of the studied compounds, defined as *I*/*I*_contr_; where *I* is the Kv1.3 current upon an application of an examined compound at +40 mV, *I*_contr_ is the Kv1.3 current recorded on the same cell at +40 mV under control conditions. For calculating the value of a half-blocking concentration (EC_50_) and the Hill’s coefficient, the magnitude of the inhibition defined as 1 − (*I*/*I*_contr_) was taken in consideration. Both parameters were calculated applying the Hill’s equation in a form: *y* = *a* + (*b* *−* a)/(1 + (*x*/*c*)^*d*^), where *y* is magnitude of the channels’ inhibition, *a* is the minimal value of the inhibitory effect, *b* is the maximal value of the inhibitory effect, *x* is the logarithm of the compound’s concentration, *c* is the logarithm of the EC_50_ value, *d* is the Hill’s coefficient. Statistical analysis was performed applying the Student’s unpaired t test. The results were considered statistically significant when *p* < 0.05.

## Results

**Fig. 2:**
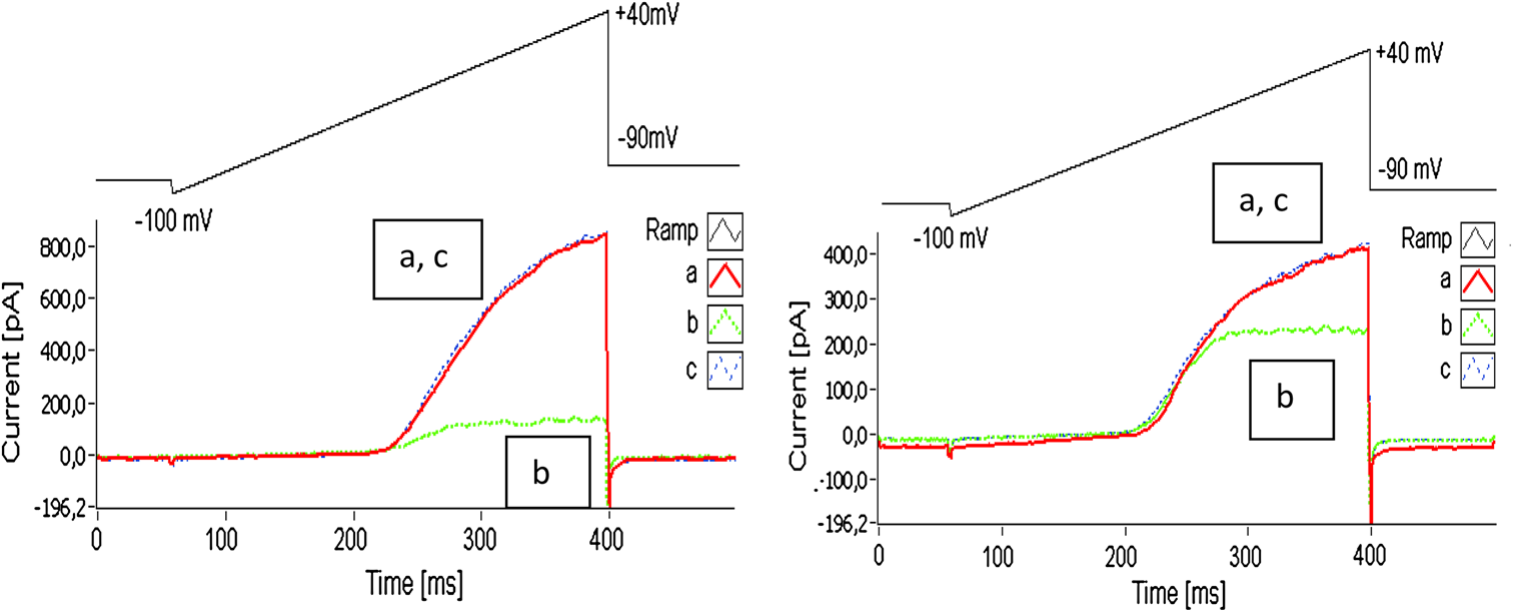
Both examined compounds reduced the intensity of the whole-cell potassium currents recorded in Jurkat T cells applying the voltage ramp protocol (shown schematically above the current records): (*a*) control conditions, (*b*) application of 15 μM of xanthohumol (*left panel*) and 10 μM of isoxanthohumol (*right panel*) and (*c*) “wash-out” of the examined compounds

Examples of the whole-cell currents recorded in Jurkat T cells under control conditions and in the presence of xanthohumol and isoxanthohumol at concentrations of 15 and 10 μM, respectively, are presented in Fig. 2 (near here).

This figure depicts the raw currents (without leak subtraction) that were recorded applying the voltage ramp protocol. The evoked currents contained two components: small linear and much bigger non-linear. The linear current was the unspecific leak current (reversal potential equal to 0 mV), whereas the non-linear component was due to activation of Kv1.3 channels (Grissmer et al. 1992; Teisseyre and Mozrzymas 2002).

Apparently, application of xanthohumol and isoxanthohumol significantly reduced the amplitude of Kv1.3 current. The currents recovered completely after wash-out of the compounds. This indicates that the inhibitory effect of tested compounds was reversible.

The inhibitory effect of the examined compounds was concentration dependent. Upon application of xanthohumol at concentrations of 1, 3, 5, 7, 10, 15, 20 and 30 μM, the relative currents (defined in “Materials and Methods” section) were reduced to 0.88 ± 0.028 (*n* = 5), 0.57 ± 0.076 (*n* = 5), 0.31 ± 0.08 (*n* = 5), 0.29 ± 0.017 (*n* = 5), 0.21 ± 0.022 (*n* = 5), 0.20 ± 0.05 (*n* = 5), 0.19 ± 0.03 (*n* = 5) and 0.14 ± 0.07 (*n* = 5), respectively. The decrease of the relative current was statistically significant (*p* < 0.05, Student’s *t* test) in case of all applied concentrations. In the case of isoxanthohumol applied at the same concentrations, the relative currents were reduced to 0.99 ± 0.068 (*n* = 5), 0.96 ± 0.077 (*n* = 5), 0.75 ± 0.028 (*n* = 5), 0.55 ± 0.047 (*n* = 5), 0.45 ± 0.059 (*n* = 5), 0.36 ± 0.071 (*n* = 5), 0.25 ± 0.091 (*n* = 5) and 0.13 ± 0.046 (*n* = 5), respectively. The decrease of the relative current was statistically significant (*p* < 0.05, Student’s *t* test) for concentrations equal or higher than 5 μM. The inhibitory effects of xanthohumol and isoxanthohumol on Kv1.3 currents in human Jurkat T cells were not complete at the highest applied concentration (30 μM).

**Fig. 3:**
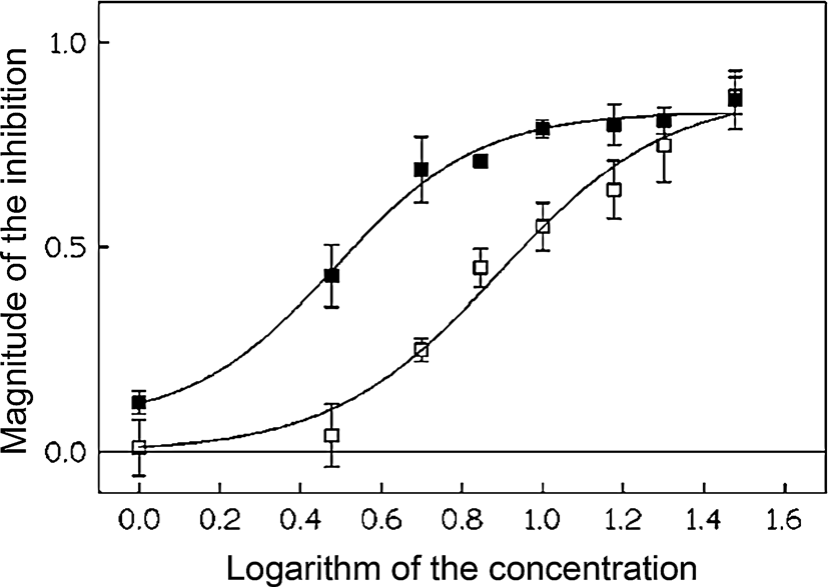
Comparison of a magnitude of the inhibition by xanthohumol (*filled squares*) and isoxanthohumol (*empty squares*) plotted as a function of logarithm of concentrations of both compounds (in μM). Data points were fitted by the Hill’s equation

In order to characterize the inhibitory effects of the compounds on Kv1.3 currents in more detail, the values of a half-blocking concentration (EC_50_) and the Hill’s coefficient were calculated, applying the Hill’s equation. The estimated EC_50_ values were about 3.06 and 7.81 μM for xanthohumol and isoxanthohumol, respectively. The difference in EC_50_ values between xanthohumol and isoxanthohumol was significant (*p* < 0.05, Student’s *t* test). This suggests that xanthohumol is much more potent inhibitor of Kv1.3 currents in human Jurkat T cells than its isomer isoxanthohumol. The estimated values of the Hill’s coefficient were 2.43 ± 0.54 and 2.09 ± 0.45 for xanthohumol and isoxanthohumol, respectively. These values were not significantly different from each other (*p* > 0.05, Student’s *t* test). Figure 3 (near here) depicts the comparison of a magnitude of the channels’ inhibition by xanthohumol and isoxanthohumol as a function of a logarithm of concentrations (in μM).

Our previous results showed that the inhibitory effect of 8-prenylnaringenin was accompanied by a significant increase in the inactivation rate of Kv1.3 currents (Gąsiorowska et al. 2012). In order to prove whether the inactivation and activation rate were changed upon an application of xanthohumol and isoxantohumol, another study applying a protocol with depolarizing voltage steps described in detail in our previous article (Gąsiorowska et al. 2012), was carried out. In contrast to what had been observed earlier for 8-prenylnaringenin, an application of xanthohumol and isoxanthohumol did not significantly change the inactivation kinetics of the currents (not shown). The activation kinetics remained unchanged upon application of the tested compounds (not shown), similarly to what had been observed earlier for 8-prenylnaringenin (Gąsiorowska et al. 2012).

## Discussion

Results of our study provide evidence that both selected compounds from the groups of prenylated chalcones and flavonoids: xanthohumol and isoxanthohumol inhibit the activity of Kv1.3 channels in human leukemic Jurkat T cells. The inhibitory effect occurred in a concentration-dependent manner. Xanthohumol is more potent inhibitor of the channels than isoxanthohumol. The channels’ inhibition was not complete at highest applied concentrations. The inhibitory effect was reversible for all the compounds tested.

The inhibitory effects of xanthohumol and isoxanthohumol on Kv1.3 channels were significantly different than the effect observed earlier for 8-prenylnaringenin (Gąsiorowska et al. 2012). First, in contrast to what was observed for 8-prenylnaringenin, the inhibitory effects of xanthohumol and isoxanthohumol were not complete at concentrations higher than 10 μM. Moreover, the inhibitory effects of these compounds were not accompanied by an increase in the currents’ inactivation rate. These results suggest that xanthohumol and isoxanthohumol are less potent inhibitors of Kv1.3 channels in human Jurkat T cells than 8-prenylnarigenin. Also the mechanism of the channels’ inhibition by these compounds is probably different.

On the other hand, the inhibitory effects of xanthohumol and isoxanthohumol on Kv1.3 channels were much more potent than the effects caused by other natural plant-derived compounds, which exert an antiproliferative effect on cancer cells, such as genistein or resveratrol (Teisseyre and Michalak 2005, 2006). Results of our previous studies provide evidence that the EC_50_ values were more than 10 μM for genistein and about 40 μM in the case of resveratrol (Teisseyre and Michalak 2005, 2006). These values were significantly higher than the EC_50_ values estimated in this study for xanthohumol and isoxanthohumol. On the other hand, it must be taken in consideration that studies on the influence of genistein and resveratrol were not performed on Jurkat T cells, but on Kv1.3 channels in normal human TL (Teisseyre and Michalak 2005, 2006). However, preliminary studies on Kv1.3 channels in Jurkat T cells demonstrated that the channels were also inhibited by resveratrol, however, the magnitude of the effect was smaller than in the case of the channels in normal human TL (Gąsiorowska—unpublished results). Therefore, the observed differences in the inhibition potency of xanthohumol, isoxanthohumol, genistein and resveratrol were not due to application of different model systems. They were probably due to an involvement of different mechanisms of Kv1.3 channel inhibition by compounds examined in this study, genistein and resveratrol.

Our earlier experiments provide evidence that a flavonoid naringenin, a precursor compound for 8-prenylnaringenin, did not inhibit Kv1.3 channels in normal human TL when applied at 30 μM concentration (Teisseyre et al. 2009). Studies performed recently on the channels expressed in cancer cells—human Jurkat T cell line—provide evidence that naringenin was not an inhibitor of Kv1.3 channels in these cells when applied at concentrations up to 30 μM (Gąsiorowska—unpublished results). The same study showed that two synthetic derivatives of naringenin: 4′,7-dimethylether and 7-methylether inhibited Kv1.3 channels in Jurkat T cells in a concentration-dependent manner (Gąsiorowska—unpublished results). The estimated EC_50_ values for the inhibition were about 13 μM and about 16 μM for naringenin-4′,7-dimethylether and naringenin-7-methylether, respectively (Gąsiorowska—unpublished results). Thus, similarly to what was observed in the case of genistein and resveratrol, the EC_50_ values estimated for both methoxy derivatives of naringenin were significantly higher than those obtained for xanthohumol and isoxanthohumol. Altogether, obtained results may indicate that prenylated flavonoids and chalcones, such as 8-prenylnaringenin, xanthohumol and isoxanthohumol, are more potent inhibitors of Kv1.3 channels than non-prenylated compounds from both groups. These results may confirm our earlier hypothesis that the presence of a prenyl group in a molecule is a factor that facilitates the inhibition of Kv1.3 channels by compounds from the groups of flavonoids and chalcones. In accordance to this hypothesis, recently performed studies with isobavachalcone—a prenylated plant-derived chalcone with the chemical structure similar to xanthohumol—showed that this compound also inhibited Kv1.3 channels in Jurkat T cells in the concentration-dependent manner. The relative current was reduced to about 0.34 at the concentration of 15 μM (Gąsiorowska—unpublished results). The estimated EC_50_ value for isobavachalcone was about 5 μM (Gąsiorowska—unpublished results).

Our results showed that the EC_50_ values for Kv1.3 channel inhibition by xanthohumol and isoxanthohumol were markedly different from each other. This may be a consequence of differences both in a chemical structure and in geometry of molecules of these compounds (Fig. 1). Interestingly, a very significant difference in the inhibition potency occurred between isoxanthohumol and 8-prenylnaringenin (Gąsiorowska et al. 2012), despite a very small difference in a chemical structure—replacement of a methoxy group in isoxanthohumol by a hydroxyl group in 8-prenylnaringenin. The inhibition of Kv1.3 channels by prenylated chalcones and flavonoids is probably a consequence of specific interactions of these compounds with the channels’ proteins. More studies are necessary to elucidate the mechanisms of the channels’ inhibition.

The inhibition of Kv1.3 channels by examined compounds may be of physiological significance. It is known that inhibition of Kv1.3 channels may also inhibit proliferation of some cell types, including breast, colon and prostate cancer cells (Comes et al. 2013; Felipe et al. 2006). It is also known that xanthohumol and isoxanthohumol inhibit cell proliferation in the case of human breast cancer cell line MCF-7, human colon cancer cell line HT-29 and ovarian cancer cell line A-2780 when applied in vitro. The EC_50_ values for the inhibition of proliferation of MCF-7 cells after 4 days of incubation were 3.47 and 4.69 μM for xanthohumol and isoxanthohumol, respectively (Miranda et al. 1999). These values are close to the EC_50_ values for the inhibition of Kv1.3 channels in Jurkat T cells. It is known that Kv1.3 channels are expressed in non-lymphocyte cancer cell lines, such as human breast cancer cell line MCF-7 (Gulbins et al. 2010). A possible contribution of the inhibition of Kv1.3 channels to antiproliferative effects of xanthohumol and isoxanthohumol might explain the observed phenomenon that MCF-7 cells were more sensitive to the flavonoids than HT-29 cells (Miranda et al. 1999). It remains to be elucidated whether inhibition of Kv1.3 channels is involved in antiproliferative effects of the examined compounds in cancer cell lines.

The inhibition of Kv1.3 channels by xanthohumol and isoxanthohumol might also be involved in proapoptotic activities of these compounds. Studies performed in the last years showed that Kv1.3 channels are expressed not only in plasma membrane but also in the inner mitochondrial membrane in normal human TL and in Jurkat T cells (Gulbins et al. 2010; Szabo et al. 2005, 2008). It is known that inhibition of mitochondrial Kv1.3 channels in human T cells by a proapoptotic protein Bax is the first crucial event in the process of an activation of the mitochondrial pathway of apoptosis (Szabo et al. 2008). In other studies, it was shown that inhibition of mitochondrial Kv1.3 channels in Jurkat T cells by membrane-permeant channel inhibitors, such as clofazimine, Psora-4 and PAP-1 mimicked the effects of Bax and triggered the apoptotic cell death by activation of the mitochondrial pathway (Leanza et al. 2012, 2013). Recently, obtained data showed that Kv1.3 channels are expressed in inner mitochondrial membrane also in non-lymphocyte cancer cells, such as human prostate cancer cell line PC-3 or breast cancer cell line MCF-7 (Gulbins et al. 2010). It is possible that the examined compounds are able to diffuse across the plasma membrane and reach intracellular compartments, so they may inhibit mitochondrial Kv1.3 channels. Delmulle et al. 2008 showed that an application of xanthohumol and isoxanthohumol at micromolar concentrations induced cell death in human prostate cancer PC-3 and DU145 cell lines. However, it was shown that in this case, the cell death occurred in a caspase-independent manner, without typical apoptotic morphological features, probably as a result of autophagy (Delmulle et al. 2008). Whether mitochondrial Kv1.3 channels are as sensitive to the inhibition by the compounds as the channels in the plasma membrane and whether this blocking effect is involved in induction of the mitochondrial pathway of cancer cell apoptosis remains to be elucidated.
